# Excess iodine exposure acutely increases salivary iodide and antimicrobial hypoiodous acid concentrations in humans

**DOI:** 10.1038/s41598-022-23803-8

**Published:** 2022-12-03

**Authors:** Yasutada Akiba, Angela M. Leung, Muhammad-Tariq Bashir, Ramin Ebrahimi, Jesse W. Currier, Natalia Neverova, Jonathan D. Kaunitz

**Affiliations:** 1 Medical Service, Section of Gastroenterology and Hepatology, Greater Los Angeles VA Healthcare System, Los Angeles, CA USA; 2 Division of Gastroenterology, Department of Medicine, David Geffen School of Medicine at UCLA, Los Angeles, CA 90095 USA; 3 Medical Service, Section of Endocrinology, Diabetes, and Metabolism, Greater Los Angeles VA Healthcare System, Los Angeles, USA; 4 Division of Endocrinology, Diabetes, and Metabolism, Department of Medicine, David Geffen School of Medicine at UCLA, Los Angeles, CA 90095 USA; 5 Medical Service, Section of Cardiology, Greater Los Angeles VA Healthcare System, Los Angeles, USA; 6 Research Service, Greater Los Angeles VA Healthcare System, Los Angeles, USA; 7 Division of Hematology and Oncology, Department of Medicine, David Geffen School of Medicine at UCLA, Los Angeles, CA 90095 USA; 8 Division of Cardiology, Department of Medicine, David Geffen School of Medicine at UCLA, Los Angeles, CA 90095 USA; 9 West Los Angeles VA Medical Center, Bldg. 114, Suite 217, 11301 Wilshire Blvd., Los Angeles, CA 90073 USA

**Keywords:** Translational research, Disease prevention, Nutrition therapy, Viral infection, Public health, Bioinorganic chemistry

## Abstract

The lactoperoxidase (LPO)-hydrogen peroxide-halides reaction (LPO system) converts iodide and thiocyanate (SCN^−^) into hypoiodous acid (HOI) and hypothiocyanite (OSCN^−^), respectively. Since this system has been implicated in defense of the airways and oropharynx from microbial invasion, in this proof-of-concept study we measured the concentrations of these analytes in human saliva from a convenience clinical sample of 40 qualifying subjects before and after acute iodine administration via the iodinated contrast medium used in coronary angiography to test the hypothesis that an iodide load increases salivary iodide and HOI concentrations. Saliva was collected and salivary iodide, SCN^−^, HOI and OSCN^−^ were measured using standard methodology. The large iodine load delivered by the angiographic dye, several 100-fold in excess of the U.S. Recommended Daily Allowance for iodine (150 µg/day), significantly increased salivary iodide and HOI levels compared with baseline levels, whereas there was no significant change in salivary SCN^−^ and OSCN^−^ levels. Iodine load and changes of salivary iodide and HOI levels were positively correlated, suggesting that higher iodide in the circulation increases iodide output and salivary HOI production. This first of its kind study suggests that a sufficient but safe iodide supplementation less than the Tolerable Upper Limit for iodine set by the U.S. Institute of Medicine (1,100 µg/day) may augment the generation of antimicrobial HOI by the salivary LPO system in concentrations sufficient to at least in theory protect the host against susceptible airborne microbial pathogens, including enveloped viruses such as coronaviruses and influenza viruses.

## Introduction

Salivary lactoperoxidase (LPO) catalyzes the oxidation of the halide iodide and pseudohalide thiocyanate (SCN^−^) to the strongly oxidant disinfectant hypohalites hypoiodous acid (HOI) and hypothiocyanite (OSCN^−^), respectively, in the presence of hydrogen peroxide (H_2_O_2_) generated by the nicotinamide adenine dinucleotide 2ʹ-phosphate (NADPH) oxidase dual oxidase (Duox). This reaction, termed the LPO system, contributes to the important anti-bacterial and anti-viral protective host defense mechanisms at the oral, respiratory, and gastrointestinal epithelial interfacial surfaces^1–3^. The LPO system is regarded as a first-line host defense mechanism against infection by airborne bacteria and viruses. Products generated by the LPO system (iodide/HOI or SCN^−^/OSCN^−^) have been suggested to have antiviral activity for enveloped viruses such as respiratory syncytial virus^4,5^, influenza viruses^6,7^, and herpes simplex virus type 1^8^. Generation of hypohalites by the LPO system is akin to the generation of hypochlorous acid (HClO) by myeloperoxidase in neutrophils with the exception that since LPO has low affinity to Cl^−^, it cannot generate HClO. Furthermore, in contrast to NADPH oxidase (Nox) in neutrophils^9^, salivary, respiratory and gastrointestinal luminal H_2_O_2_ is supplied by other members of Nox family, namely Duox; the airway epithelium predominantly expresses Duox1^2^, whereas salivary ductal and intestinal epithelia express Duox2^10,11^. Iodide and SCN^−^, the primary LPO substrates, are taken up from the bloodstream via the sodium iodide symporter (NIS; SLC5A5) at the basolateral membrane of salivary duct cells and gastric epithelial cells as well as the thyroid follicles^12^, followed by the secretion into the saliva or gastric lumen through the anion channels cystic fibrosis transmembrane conductance regulator (CFTR) or anoctamin-1 (ANO1, also known as transmembrane member 16A; TMEM16A), or the anion exchanger pendrin (SLC26A4) at the apical membrane^13^.

Prevention of infection from airborne enveloped viruses such as Severe Acute Respiratory Syndrome Coronavirus 2 (SARS-CoV-2) have consisted primarily of mass vaccination and public health measures such as masking, social distancing, and quarantines. A seldom-discussed intervention is strengthening the aforementioned LPO system in the oropharynx with supplemental dietary iodine, in particular derived from marine plants such as kelp and seaweed^14^. Detailed studies have reported that salivary iodine concentrations and salivary secretion rates are highly correlated with dietary iodine consumption with a linear correlation when consumption is < 167 µg/day, and an exaggerated, nonlinear relation when consumption exceeds that level^15^, suggesting that iodide levels are concentrated in the saliva at high levels of serum iodide, although the majority (~ 90%) of iodine taken in is renally excreted. It is plausible that by increasing daily iodine intake from the level guided by the U.S. Recommended Daily Allowance (RDA; 150 µg/day) to the U.S. Institute of Medicine’s Tolerable Upper Limit (UL) of 1100 µg/day^16^, a 1100/150 (7.3-fold) increase, the higher iodine dose would augment salivary iodine (and presumably HOI) concentrations by a comparable amount. Furthermore, the UL of iodine consumption in some diets may be adequate to produce viral suppressive HOI concentrations in the saliva.

Despite the theoretical possibility of bolstering this endogenous antiviral system through increasing dietary iodine consumption, salivary levels of bioactive products of the salivary LPO system have not been studied in humans. We hypothesize that an excess iodide load to the bloodstream may, through increasing salivary iodide secretion, increase the salivary concentration of antimicrobial products of the LPO system in humans. Thus, we examined the salivary levels of iodide, SCN^−^, HOI and OSCN^−^ after iodinated contrast administration routinely used in patients undergoing coronary angiography.

## Materials and methods

### Human subjects

We used a convenience sample of 49 subjects, consecutively enrolled from August 20, 2020 to September 9, 2021, selected from Veterans undergoing coronary catheterization as part of their routine medical care. Inclusion criterion: Subjects who had been scheduled to undergo coronary angiography as part of usual care at the West Los Angeles Veterans Affairs Medical Center were enrolled into the study. Exclusion criteria: (1) refusal to be included in the study; (2) inability to provide informed consent; and (3) inability or refusal to provide an adequate saliva sample. Data analysis was performed on only the subset of 40 subjects who provided the baseline saliva sample and at least one post-iodinated contrast exposure salivary specimen of sufficient volume for the laboratory measurements.

All subjects had a negative COVID-19 PCR test confirmed before coronary angiography. During catheterization for coronary angiography, 25–230 ml of iodinated contrast medium (Visipaque®-320) was injected intraarterially so as to visualize the coronary arteries. In study 1 (n = 30), saliva was collected at two timepoints: immediately before and 6 h after iodinated contrast exposure; 6 subjects were excluded due to insufficient saliva volume. In study 2 (n = 19), saliva was collected at up to 5 timepoints: immediately before and at 6, 24, 30 and 48 h after iodinated contrast exposure; 3 subjects were excluded due to insufficient saliva or cancellation of the coronary angiography procedure. Thus, of the 49 subjects enrolled, 9 were excluded with 40 analyzed: 24 subjects in study 1 and 16 subjects in study 2.

There was no difference in the iodine load using Visipaque-320 that contains iodine 320 mg/ml, between study 1 and study 2; mean (SD) of iodine load was 27.6 (18.6) g (n = 24) in study 1 and 19.0 (8.3) g (n = 15) in study 2 (p = 0.2368, Mann–Whitney test). In order to minimally disrupt the subject’s medical care, saliva sampling at the 6 h point was variable. The calculated time-point mean (SD) was 5.9 (1.1) h (n = 24) in study 1 and 5.7 (1.2) h (n = 16) in study 2 (p = 0.5511, Mann–Whitney test), respectively. Therefore, we used ‘6 h’ for the second saliva sample. In contrast, sampling 24, 30, and 48 h time points was carried out with much greater precision. Among 16 cases in study 2, sampling at 0–6-24 h was performed in 12 cases, with collections at 0–6-24–48 h in 1 case, and at 0–6-24–30-48 h in 3 cases. Therefore, for statistical consistency, we analyzed the 16 cases that completed collections at 0–6-24 h. For 48 h, 4 cases in study 2 were analyzed.

### Saliva collection

Saliva was collected using a universal saliva collection kit (Super SAL, Oasis Diagnosis, Vancouver, WA, USA). All subjects were fasted, generally after midnight prior to the catheterization procedure. Prior to saliva collection, the subjects rinsed their mouths with plain water which was then expectorated. Five min after the oral rinse, they were given illustrated instructions on how to collect saliva with the Super SAL device: https://oasisd.wpengine.com/wp-content/uploads/2018/07/Super%E2%80%A2SAL%E2%84%A2-SBS-5-10-17.pdf that are available in English or Spanish. The saliva was collected until the appearance of the sample volume adequacy indicator changed completely to red. The saliva was transferred into an Eppendorf tube. This procedure was repeated until saliva volume reached ~ 1 ml. The collection procedure generally lasted 5–10 min. The second saliva sample was collected 6 h after cardiac catheterization as described above. In study 2, saliva samples were also collected 24, 30 and 48 h after catheterization as described above. Most of subjects had lunch and breakfast before sampling at 6 and 30 h, and at 24 and 48 h after catheterization, respectively. The meals consisted of a standard hospital food with mean estimated content of iodine 41 µg/meal^17^, but with unknown thiocyanate content. The timepoints for collection were determined by subjects’ availability and ability to provide a saliva sample before and in the first 48 h after their cardiac catheterization procedure as clinically indicated; in order to minimize disruption to their usual medical care, subjects were approached for repeat salivary sampling as determined by their clinical state and willingness to participate within those 48 h, with up to a maximum of 4 collections possible post-catheterization.

Saliva specimens were stored at 4 °C and centrifuged at 1000×*g* for 5 min on the day of each collection in order to remove cellular debris and other sediment. The supernatant was stored at – 20 °C until analysis. Some samples contained viscous protein aggregates likely due to mucus in the thawed saliva samples which was separated from the liquid phase prior to analysis.

### Regulatory compliance

The study was performed with approval of the Greater Los Angeles Veterans Affairs Healthcare System Institutional Review Board (IRB) and is compliant with all institutional policies covering human research. Written informed consent was obtained from each enrolled subject. This research conformed to the standards set by the latest version of the *Declaration of Helsinki* or the version that was in place at the time of the experiments. Note that since the sole intervention used for the research was the collection of saliva, this study was considered to be “minimal risk”. Under the study terms dictated by the IRB, no patient-level descriptive data such as age, sex, gender, diagnosis, medications, comorbidities, smoking history, or any other clinical data were recorded.

### Chemicals

Ionic strength adjustors (ISA) solution for iodide was purchased from Thermo Fisher Scientific (#940011, Waltham, MA, USA). Sodium iodide (NaI), iodine, sodium thiocyanate (NaSCN), lactoperoxidase (LPO), 5,5’-dithiobis-(2-nitrobenzoic acid) (DTNB) and NADPH tetrasodium salt hydrate (β-NADPH), ferric chloride (FeCl_3_), hydrogen peroxide (H_2_O_2_) and other chemicals were purchased from Sigma Chemical (St. Louis, MO, USA). Iodine was freshly dissolved in 95% ethanol. All other solutions were made with Milli-Q water.

### Laboratory measurements

#### Iodide

The iodide content in the saliva was measured using an Orion iodide electrode (Thermo Fisher Scientific). The iodide selective electrode was selective for iodide in the range of 1 µM–1 mM over SCN^−^ or Cl^−^. A standard curve for NaI (1 µM–1 mM) in 10% ISA solution was made every time. Fifty µl of the saliva sample was added to 450 µl of 10% ISA solution and then mV was read after stabilization.

#### SCN^−^

The SCN^−^ content in the saliva was measured by ferric colorization. Twenty-five µl of NaSCN standard (10 µM–2.5 mM) in Tris buffer (50 mM, pH 7.4) or samples was placed in a clear flat-bottom 384-well plate (Nunc, Thermo Fisher Scientific) and 25 µl of FeCl_3_ (0.1 M) in Tris buffer was added into each well. The absorbance at 490 nm was read using a multi-mode microplate reader (Synergy-2, BioTek Instruments, Inc., Winooski, VT, USA). The yellow color of FeCl_3_ will be immediately turns to deep red according to the production of [Fe(SCN)]^2+^. Grossly reddish saliva samples with high background absorbance at 490 nm were omitted from the analysis due the high likelihood of inaccurate measurements.

#### OSCN^−^

The OSCN^−^ content in the saliva was measured using Ellman’s reagent according to the previous report^18^. DTNB 10 mM 6.4 µl was added to Tris buffer (992.6 µl, 50 mM, pH 7.4), followed by the addition of 1-µl of 2-mercaptoethanol (60 mM) to make yellow TNB solution (60 µM). SCN^−^ standard (2 µM–2 mM) was made from 1 M NaSCN stock solution in Tris buffer. LPO 100 µg/ml 40 µl and H_2_O_2_ 10 mM 8 µl were added in Tris buffer total 1 ml to make the reaction solution containing LPO 4 µg/ml and H_2_O_2_ 80 µM. Equal volume of SCN^−^ standard and the reaction solution was mixed to generate OSCN^−^ standard solution. The chemical reaction is shown below:

$${\text{SCN}}^{ - } + {\text{H}}_{2} {\text{O}}_{2} \mathop{\longrightarrow}\limits^{{{\text{LPO}}}}{\text{OSCN}}^{ - } + {\text{H}}_{2} {\text{O}}$$

Twenty-five µl of OSCN^−^ standard or samples was placed in a clear flat-bottom 384-well plate and 25-µl of TNB solution freshly prepared was added to each well. The absorbance at 412 nm was read for 10 min with 2-min interval using Synergy-2. The yellow color of TNB solution was diminished by the oxidation of TNB to DTNB (colorless) by the presence of OSCN^−^ in the standard and samples. Background absorbance of samples at 412 nm was also measured separately and subtracted from the OSCN^−^ measured values.

#### HOI

The HOI content in the saliva was measured using NADPH conversion to iodinated NADPH (NADPI) according to the previous report^19^. In situ production of HOI from NaI with LPO and H_2_O_2_ was possible but with a lower conversion rate. NADPI was freshly prepared as previously reported^20^ and used as chemical standard. The chemical reactions are shown below:

$$\begin{gathered} {\text{I}}^{ - } + {\text{H}}_{2} {\text{O}}_{2} \mathop{\longrightarrow}\limits^{{{\text{LPO}}}}{\text{HOI}} + {\text{OH}}^{ - } \hfill \\ {\text{NADPH}} + {\text{HOI}} \to {\text{NADPI}} + {\text{H}}_{2} {\text{O}} \hfill \\ \end{gathered}$$

Ten µl of iodine solution (10 mM in 95% ethanol) and 1 µl of NADPH (0.1 M) were added to Tris buffer to make 1 ml of 100 µM NADPI solution. The absorbance of NADPH at 340 nm was rapidly bleached by the addition of iodine, with complete conversion of NADPH to NADPI. The samples (25 µl) were reacted with 25 µl of 500 µM NADPH in Tris buffer in a clear flat-bottom 384-well plate. The absorbances of blank (Tris buffer only), NADPI standards and the reacted samples at 340 nm for NADPH and 282 nm for NADPI were read for 20 min at 2-min intervals using the Synergy-2 instrument. According to HOI content, the 340 nm reading (NADPH) was reduced, whereas the 282 nm reading (NADPI) was increased. Background absorbance of samples at 340 and 282 nm was also measured separately and subtracted from the HOI measured values. Increased absorbance at 282 nm was used to calculate the NADPI content, equivalent to the HOI content in the samples.

### Statistical analysis

Sample size was determined according to the results of a preliminary study of 10 subjects, in which we found a ~ 2.5-fold increase in salivary iodine levels after catheterization with a ~ 50% standard deviation, yielding predicted minimal sample sizes of 12 (80% power) and 16 (90% power) at α = 0.05. Values are individually plotted as before and after pairs and expressed as median [interquartile range (IQR)]. Statistical analysis was performed with GraphPad® Prism 9.4.1 (La Jolla, CA, U.S.A.; https://www.graphpad.com) using Wilcoxon matched-pairs signed rank test, two-tailed, or Friedman test, two-tailed, followed by Dunn’s multiple comparison test. Correlation between iodide content in the injected contrast medium and the changes in iodide levels in saliva, or between the levels of iodide and HOI, was assessed by simple linear regression and Spearman correlation. Differences were considered significant when *P* values were < 0.05.

### Ethical approval

Institutional Ethics Committee Approval number: iRIS# 2020-000172; ePromise# 0027-1221594; IRBNet # 1616067.

## Results

### Salivary contents of iodide, SCN^−^, HOI and OSCN^−^ before and after iodinated contrast exposure

**Figure 1:**
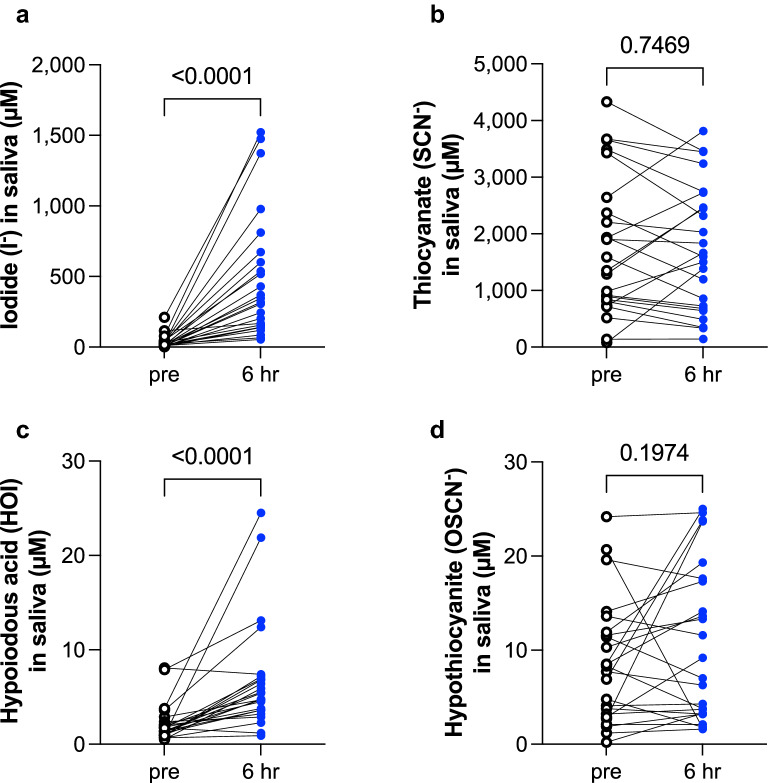
Study 1: Salivary contents of iodide, thiocyanate (SCN^−^), hypoiodous acid (HOI) and hypothiocyanite (OSCN^−^) before and 6 h after an iodinated contrast load. Saliva was collected before (pre) and 6 h after an iodinated contrast load. Salivary levels of iodide (**a**), SCN^−^ (**b**), HOI (**c**), and OSCN^−^ (**d**) were measured as indicated in Methods. All data were analyzed by paired Wilcoxon matched-pairs signed rank test, two-tailed (n = 24 pairs). P values shown above brackets.

In study 1, salivary iodide concentrations (µM) were increased after iodinated contrast exposure (median [IQR]; pre 16.0 [9.6–35.7], 6 h 331.9 [145.5–654.7], p < 0.0001) (Fig. 1a). In parallel, salivary HOI concentrations (µM) were increased after iodinated contrast exposure (pre 1.6 [0.9–2.3], 6 h 5.1 [3.6–7.1], p < 0.0001) (Fig. 1c). Conversely, there were no changes in salivary levels of SCN^−^ (µM) and OSCN^−^ (µM) before and after iodinated contrast exposure; SCN^−^, pre 1471 [815.1–2575], 6 h 1633 [693.3–2661], p = 0.7469; OSCN^−^, pre 8.0 [3.0–11.8], 6 h 8.1 [3.2–17.5], p = 0.1974 (Fig. 1b,d).

**Figure 2:**
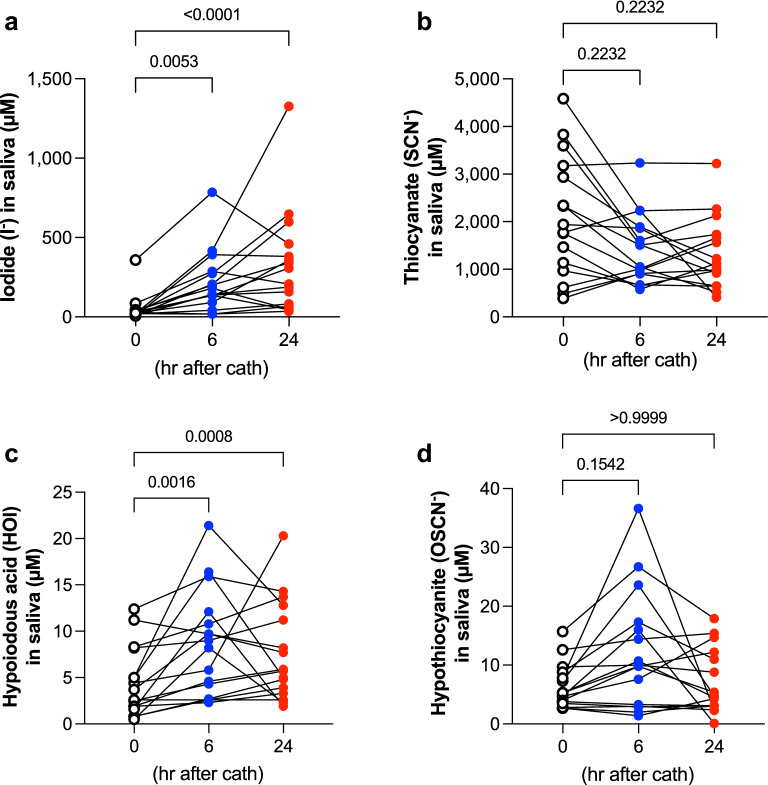
Study 2: Salivary contents of iodide, thiocyanate (SCN^−^), hypoiodous acid (HOI) and hypothiocyanite (OSCN^−^) before, 6 h and 24 h after an iodinated contrast load. Saliva was collected before (0 h), 6 h and 24 h after an iodinated contrast load. Salivary levels of iodide (**a**), SCN^−^ (**b**), HOI (**c**), and OSCN^−^ (**d**) were measured as indicated in Methods. All data were analyzed by Friedman test, two-tailed, followed by Dunn’s multiple comparison test (n = 16 pairs). P values vs. 0 h shown above brackets.

In study 2, salivary iodide concentrations were increased over time following iodinated contrast exposure (0 h 24.3 [19.6–36.0], 6 h 161.1 [102.0–283.4], 24 h 256.6 [70.6–440.0]) (Fig. 2a). Salivary levels of HOI after iodinated contrast exposure was also higher than the basal level at 0 h (0 h 3.2 [1.7–7.4], 6 h 8.6 [3.1–11.8], 24 h 5.8 [3.5–12.4]) (Fig. 2c). In contrast, there were no changes in salivary levels of SCN^-^ and OSCN^−^ before and after iodinated contrast exposure; SCN^−^, 0 h 1854 [1010–3117], 6 h 1281 [905.0–1883], 24 h 1081 [717.1–1715]; OSCN^−^, 0 h 5.0 [3.6–8.6], 6 h 9.9 [3.1–17.0], 24 h 4.7 [3.1–11.9] (Fig. 2b,d). In 4 subjects, saliva was collected up to 48 h; salivary iodide levels at 48 h were higher than the basal levels, but not significant (0 h 19.9 [16.9–20.9], 48 h 68.1 [43.5–861.7], p = 0.125).

Between studies 1 and 2, pre iodide levels were statistically similar, whereas 6 h iodide levels were significantly higher in study 1 (Table 1). Pre HOI levels were significantly higher in study 2, with no significant difference at 6 h. These differences were possibly due to subject variables in two cohorts.

**Table 1:** Comparison of studies 1 and 2 in terms of salivary iodide and HOI levels before (pre) and 6 h after iodine contrast injection.

Collection time	Salivary Iodide (µM)			Salivary HOI (µM)		
	Study 1	Study 2	p	Study 1	Study 2	p
pre	16.0 (9.6–35.4)	24.3 (19.6–36.0)	0.225	1.6 (0.9–2.3)	3.2 (1.7–7.4)	0.029
6 h	331.9 (145.5–654.7)	161.1 (102.0–283.4)	0.027	5.1 (3.6–7.1)	8.6 (3.1–11.8)	0.321

Data are expressed as median (IQR), analyzed by Mann–Whitney test.

### Correlations between iodinated contrast load, salivary levels of iodide, and salivary HOI levels

**Figure 3:**
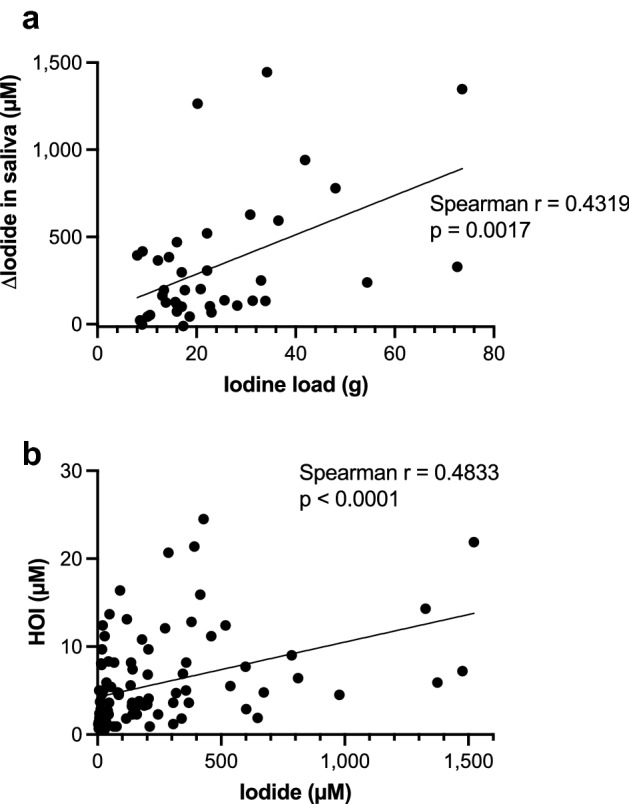
Correlations between iodine load, and salivary iodide and HOI levels. (**a**) Paired iodine content in the injected contrast medium (iodine load) and the changes in iodide levels (Δiodide) in saliva from all data of baseline and 6 h after the injection were plotted (n = 39 pairs). Correlation between the iodine load and Δiodide in saliva was analyzed by the Spearman correlation test and r value, with a simple linear regression fitted line and p value added to the graph. (**b**) Paired iodide and HOI values in saliva from all data were plotted (n = 96 pairs). Correlation between iodide and HOI levels was analyzed by the Spearman correlation test and r value, with a simple linear regression fitted line and p value added to the graph.

We analyzed the correlations between the iodine load (g), as calculated from the iodine content (320 mg/ml) and volume used (ml) of the contrast medium, and the changes in salivary iodide levels (Δiodide, µM) before and at 6 h (overall time-point mean ± SD, 5.8 ± 1.1 h) after iodinated contrast exposure from both studies (n = 39 pairs with one missing contrast volume information). Δiodide values in the saliva were positively correlated with the iodine load (simple linear regression; R^2^ = 0.2369, p = 0.0017, Spearman correlation; r = 0.4319, p = 0.0060) (Fig. 3a), suggesting that higher free iodide levels in the circulation increases salivary iodide output.

We also plotted salivary levels of iodide versus HOI of all data from both studies (n = 96 pairs). Salivary HOI levels were positively correlated with salivary iodide levels (simple linear regression; R^2^ = 0.1542, p < 0.0001, Spearman correlation; r = 0.4833, p < 0.0001) (Fig. 3b), suggesting that the increased iodide output in saliva enhances the production of HOI in saliva.

## Discussion

This is the first report of the effects of an excess iodide load on the salivary output of the halide iodide, the pseudo-halide SCN^−^, and the corresponding antimicrobial oxidants HOI and OSCN^−^ in humans.

Though iodine is a micronutrient needed for the synthesis of thyroid hormone^21^, iodine uptake also occurs in the lactating mammary gland, gastric mucosa, and salivary glands^22^, although the functional purpose of iodine uptake in the latter two tissues remains poorly understood. A single dose of iodinated contrast supplies several 100-fold the RDA of iodine (150 µg iodine per day; also see discussion below) needed for thyroid hormone production^23^. The present results suggest that an excess iodide load acutely increases salivary iodide output and enhances salivary HOI concentrations.

The SARS-CoV-2 virus infects and replicates in a subpopulation of oral cells and salivary gland cells that express the ectoenzymes angiotensin converting enzyme 2 (ACE2) and transmembrane protease, serine 2 (TMPRSS2)^24^. Saliva from asymptomatic patients with COVID-19 contains infectious virus^24^, suggesting that aerosolized saliva is the source of airborne infection of SARS-CoV-2, and that saliva is a potential therapeutic target for the treatment and prevention of COVID-19. Oral rinses or antimicrobial mouthwash formulations containing ethanol, hydrogen peroxide and povidone-iodine have been proposed for preventing transmission of SARS-CoV-2^25,26^. A recent narrative review has summarized the potential applications of these agents to the oropharyngeal space in the efforts to reduce SARS-CoV-2 viral load, particularly in high-exposure settings^27^. Nevertheless, the effects of these interventions are discontinuous, thus requiring a sustainable supply of antimicrobial compounds in order to prevent transmission of and infection with SARS-CoV-2. Our data showed that higher levels of iodide and HOI than baseline were present in the saliva at least for 24 h after systemic iodine administration. Enhanced and sustained output of endogenously generated antimicrobial oxidant species, such as HOI and OSCN^−^, appear to be better suited for this purpose.

Salivary iodine is one of several antioxidant electron donors that consumes H_2_O_2_ with LPO, generating HOI. We speculate that the depletion of H_2_O_2_ and generation of HOI, both highly reactive oxygen donors, contributes to oral cavity health and homeostasis as previously described^28^. In addition of superoxide dismutase and catalase, salivary LPO is an antioxidant enzyme that contributes to the homeostasis of the organs in which it is expressed^29^. Global LPO deficiency causes multiple organ inflammation and induces tumors^30^, suggesting that LPO and its primary oxidant products (OSCN^−^ and HOI) are part of an essential anti-inflammatory and antitumor protective mechanism in vivo.

The antimicrobial effects of hypohalous acids such as HOI on enveloped viruses is variable and unpredictable. Although the few in vivo experiments of iodine supplementation in sheep or cattle infected with respiratory viruses have yielded promising results, the pathogenic microorganisms tested did not include coronaviruses^5,31,32^. Patel et al. measured a half-maximal inhibitory concentration (IC_50_) of iodide ~ 10 µM with complete inhibition > 100 µM for influenza viruses in vitro^6^, suggesting that a concentration of 10 µM or less is close to the IC_50_ for HOI, dependent on conversion rate of HOI production from iodide and H_2_O_2_ with LPO. Since HOI production is dependent on H_2_O_2_ availability, it is difficult to determine the conversion rate of iodide to HOI. The overall salivary iodide levels pre 22.4 [10.7–36.7] (median [IQR]) and 6 h 225.1 [136.6–495.4] µM, and HOI levels pre 1.9 [1.1–3.8] and 6 h 5.8 [3.6–10.5] µM calculated from the combined data in study 1 and 2 suggest that the predicted conversion rates are 8.5% and 2.6%, respectively. Since 100 µM iodide in cell-free assay system completely inactivates H1N2 influenza virus^6^, this suggests that HOI concentrations of 2.6–8.5 µM may be in the effective range for viral inactivation, consistent with our reported mean HOI levels of 5.8 [3.6–10.5] µM at 6 h after iodinated contrast exposure, indicating the possible viricidal range of HOI in the saliva following an iodide load. Furthermore, HOI was viricidal for SARS-CoV at unstated concentrations in another study^33^. We thus will consider ~ 5 µM HOI as a reasonable theoretical concentration of HOI for inhibition of coronaviruses. Moreover, the higher levels of salivary HOI were sustained for at least 24 h after iodinated contrast exposure and likely up to 48 h, suggesting that a single excess iodide load may be enough to elevate HOI levels in the saliva for several days. Although a statistically significantly positive correlation was observed between salivary iodide and HOI levels, the correlation coefficient was modest likely reflecting background variation among the subjects.

One possible rate-limiting step of HOI production is H_2_O_2_ production in the saliva. H_2_O_2_ is presumably provided by the salivary ductal Duox2^10^, although Duox2 expression and activity may be independent variables. In preliminary studies, we also measured H_2_O_2_ levels in the saliva in some samples that were detectable at low level (< 0.1 µM; data not shown), suggesting that most H_2_O_2_ was already consumed to produce HOI and OSCN^−^. Therefore, modulating the levels of Duox2 activity and inducing its expression may be the key factors determining antimicrobial oxidant production by the LPO system.

Another hypohalite, OSCN^−^, has been extensively studied as an antimicrobial oxidant. The reported baseline salivary levels of OSCN^−^ in 40 donors were 9.73 ± 6.62 µM (mean ± SD) measured by the most recent techniques^34^. Our study showed that overall OSCN^−^ levels were 9.05 ± 7.34 µM (mean ± SD), consistent with the previous report. Possible viricidal activity of OSCN^−^ for SARS-CoV-2 has been reported in vitro with IC_50_ ~ 11 µM^35^, suggesting that salivary OSCN^−^ levels likely attain viricidal levels at baseline. Higher SCN^−^ intake may produce higher salivary SCN^−^ output and OSCN^−^ production, which may affect SARS-CoV-2 transmission, according to epidemiologic observations linking the per-capita national ingestion of cassava^36,37^ with COVID-19 mortality rates for different countries, and cigarette smoking with SAR-CoV-2 infectivity rates^38,39^, the latter population which has documented high circulating and salivary SCN^−^ levels due to the HCN content of tobacco smoke^40^. Further study is necessary to compare the effects of OSCN^−^ and HOI on SARS-CoV-2 viability.

Our data provide insight into the approximate kinetics of injected iodinated organic compounds. Generally, non-ionic iodinated contrast media contain 320–370 mg/ml of iodine as organically-bound iodine, as well as free inorganic iodide. The upper limit of free iodide in injected contrast media is < 50 µg/ml, as stipulated by production regulations. Rendl et al. reported that since most formulations of modern, non-ionic contrast media contain 0.5–2.5 µg/ml free iodide^41^, a single injection of 100 ml contrast medium results in 50–250 µg free iodide released intravascularly. Nevertheless, a significant, rapid increase of plasma iodide levels exceeds the initial free iodide content in contrast medium, suggesting that in vivo enzymatic deiodination of organically-bound iodine in contrast medium releases free iodide into the circulation. Intravenous injection of 85 ml contrast medium (300 mg/ml iodine) with the addition of 2 or 5 µg/ml free iodide rapidly increases plasma levels of inorganic iodide to 10–15 µg/dl at ~ 3 min and further to ~ 20 µg/dl at 60 min, whereas injection of saline with 2 or 5 µg/ml free iodide alone reaches only ~ 2.5 µg/dl, suggesting that free iodide is rapidly released from organic molecules by presumed enzymatic deiodination^41^. Our data showed that overall salivary iodide levels after iodinated contrast exposure was 225.1 [136.6–495.4] µM (median [IQR]), compared with the median baseline level of 22.4 [10.7–36.7] µM. Based on current understanding^41^, plasma levels of iodide in our study after iodinated contrast exposure are estimated to be 20 µg/dl, equivalent to 1.58 µM, thus representing a 143-fold higher iodide concentration in saliva than plasma, most likely due to the concentrative effect of NIS localized to the basolateral membranes of the salivary intercalated and striated duct cells in humans^10,42^. Due to competition between iodine and SCN^−^ for transport by the NIS^12^, we hypothesized that an exogenous iodine load would decrease SCN^−^ output and OSCN^−^ production, a prediction not supported by the data (Fig. 1b,d). Furthermore, our data showing that the higher iodine load in the intravascularly administered contrast medium proportionally increased the salivary iodide output. These results suggest that salivary iodide levels are non-invasively obtained biomarkers for plasma iodide levels after excess iodine exposure, since the proportional relationship between plasma iodide and salivary iodide levels has been supported by us and others^43,44^.

Although the population-wide use of iodine supplements is an attractive proposition, exposure to excess iodine, even as a single instance, has the potential to result in iodine-induced thyroid dysfunction that may in turn lead to end-organ damage^45^. The U.S. Institute of Medicine advises a Tolerable Upper Limit of 1,100 µg of iodine per day^16^, for which the American Thyroid Association recommends against the ingestion of any iodine supplements containing > 500 µg iodine per daily dose^46^. Our study measured the levels of chemical species in the LPO system before and after iodinated contrast exposure, at levels of iodine exposure that even in routine medical settings have the potential of inducing thyroid dysfunction^47–50^. Furthermore, the effects of excess iodine, including altered immune-cytotoxicity and male reproduction, have been described^51,52^. The effects of oral iodide supplementation with potassium iodide tablets or iodide-containing solutions, at iodine doses not exceeding the Tolerable Upper Limit^16^ on the salivary LPO system should be studied.

Several limitations in the present study exist. First, the sample size was small. Second, the present study was strictly observational and as a pilot proof-of-concept design, mechanistic analysis was limited. Third, detailed patient-level information of the subjects was not available, including cigarette smoking history, which is important due to the aforementioned contribution of smoking to elevated salivary SCN^−^ concentrations. Fourth, no data regarding the effect of the iodine load and salivary hypohalite concentrations on viral infection or transmission was obtained. Fifth, the colorimetric assays used to measure salivary halides and hypohalites can be subject to confounding factors, although this was addressed in the methodology. Finally, although the saliva collection was performed in accordance with the manufacturer’s instructions, there are possible differences in technique related to any recent gargling with antimicrobial solutions, recent food intake, presence of dental caries, and use of dentures. Since the saliva collections were periprocedural, all were obtained in fasting subjects at baseline. Note that the subjects took regular hospital meals containing a mean 41 µg/meal of iodine to meet U.S. RDA for iodine, that might not affect the salivary levels of iodide and HOI after iodinated contrast exposure. The individual variations in salivary gland NIS expression levels, H_2_O_2_ production by Duox2 expression, and oral cavity conditions may cause the differences in levels of iodide and HOI between study 1 and 2. Although limited data^53^ support that these factors may affect baseline salivary hypohalite concentrations and as seen from the variable baselines in our data, the use of paired data analysis reasonably controls for this variation since every subject serves as their own control regardless of baseline values.

In conclusion, excess iodide load rapidly increases salivary output of iodide and HOI that sustains up to 24 h. There is sufficient biological plausibility to justify the further study of this putatively endogenous, inexpensive, sustainable, non-immunologic antimicrobial defense mechanism in the oral cavity.

## Data Availability

All data are described in the text and depicted in the figures.

## Abbreviations

SCN^−^: Thiocyanate
OSCN^−^: Hypothiocyanite
HOI: Hypoiodous acid
NIS: Sodium iodide symporter
LPO: Lactoperoxidase
DTNB: 5,5’-Dithiobis-(2-nitrobenzoic acid)
β-NADPH: β-Nicotinamide adenine dinucleotide 2’-phosphate reduced tetrasodium salt hydrate
NADPI: Iodinated NADPH
RDA: Recommended daily allowance
UL: Tolerable upper limit
